# Engineering graphene oxide interfaces for electrochemical biosensing of biomolecules, cells, and organoids

**DOI:** 10.1186/s40580-026-00560-2

**Published:** 2026-06-30

**Authors:** Huijung Kim, Cheol-Hwi Kim, Chang-Dae Kim, Zhengtang Luo, Tae-Hyung Kim

**Affiliations:** 1 Department of Intelligent Precision Healthcare Convergence, Institute for Cross-disciplinary Studies (ICS), Sungkyunkwan University (SKKU), Seobu- ro, Jangan-gu, Suwon, 16419 Republic of Korea; 2 Department of Chemical and Biological Engineering, The Hong Kong University of Science and Technology, Kowloon, 999077 Hong Kong People’s Republic of China; 3 Department of Biomedical Engineering, ICS, SKKU, Seobu-ro, Jangan-gu, Suwon, 16419 Republic of Korea; 4 Department of MetaBioHealth, SKKU, Seobu-ro, Jangan-gu, Suwon, 16419 Republic of Korea

**Keywords:** Graphene oxide, 2D materials, Electrochemical biosensor, Surface functionalization, Biomedical sensing

## Abstract

Graphene oxide (GO) has established itself as a premier material for electrochemical biosensing due to its exceptional chemical tunability, aqueous processability, and unique sp²-sp³ hybridized structure. This review provides a comprehensive analysis of diverse engineering strategies to functionalize GO, enabling highly sensitive and selective detection of a broad spectrum of biological analytes. We systematically categorize these advancements into five key methodologies: (1) controlled reduction to precisely tune electrical conductivity and surface defects, (2) covalent functionalization for robust bioreceptor immobilization, (3) non-covalent modification to preserve biomolecular conformation, (4) metal nanoparticle hybridization for enhanced electrocatalysis, and (5) integration with polymeric/framework materials to build advanced three-dimensional sensing architectures. By examining applications ranging from small molecule metabolites and proteins to nucleic acids and whole pathogens, we demonstrate how tailored GO interfaces overcome conventional sensing trade-offs. Finally, we highlight the pivotal role of these engineered GO platforms in addressing the challenges of real-time monitoring at complex biological interfaces, including living cells and organoids, and outline the pathway toward clinically deployable diagnostic technologies.

**Graphical abstract:**

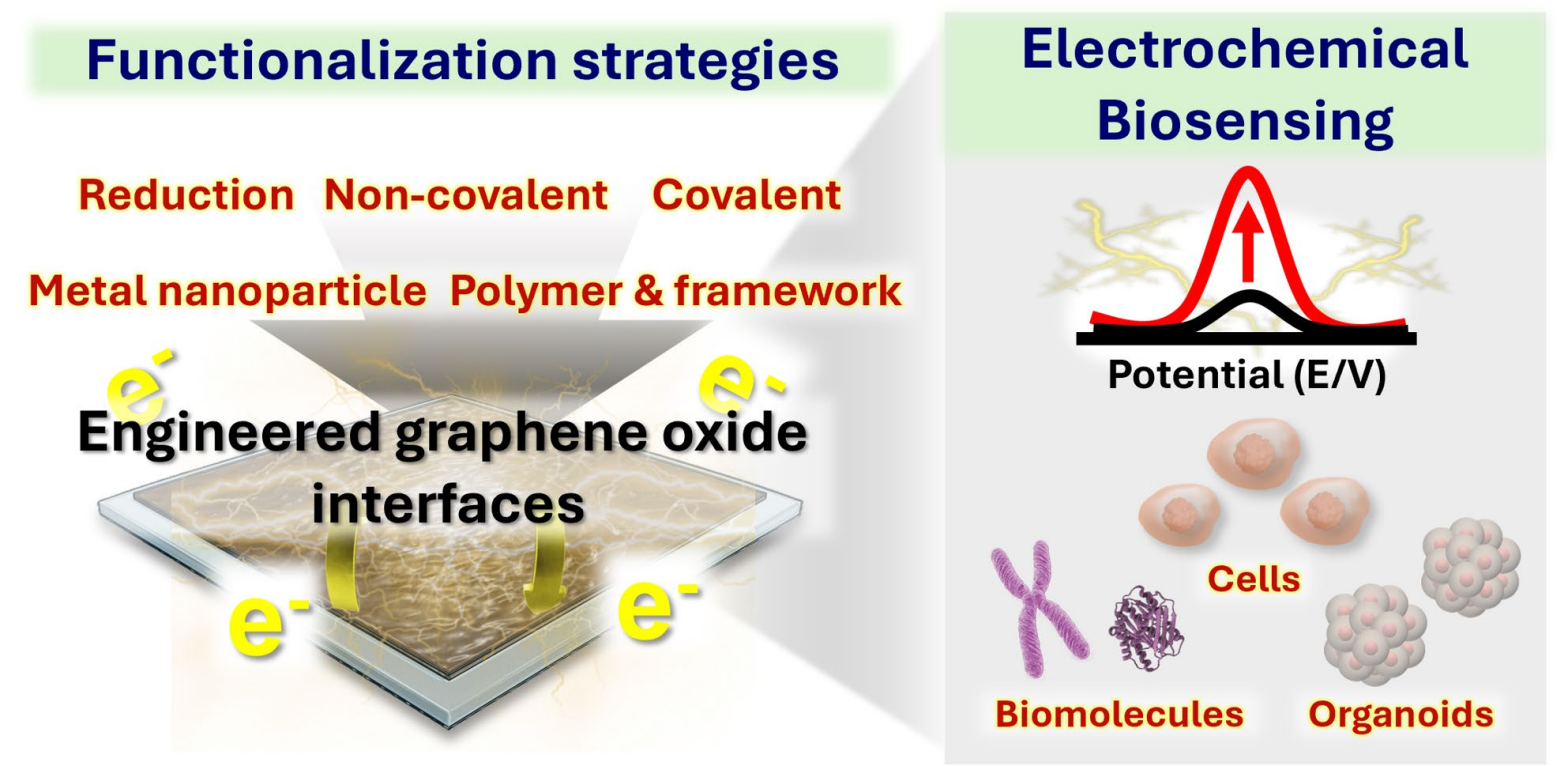

## Introduction

Graphene oxide (GO) has emerged as a transformative platform at the intersection of nanotechnology and electro-diagnostics, owing to its unparalleled chemical flexibility and unique sp²*-*sp³ hybridized framework [1–8]. Unlike pristine graphene, which consists of a perfect two-dimensional honeycomb lattice of sp² bonded carbon atoms, the structural heterogeneity of GO—characterized by a rich landscape of oxygenated functional groups—enables precise modulation of both electronic properties and interfacial chemistry [9–14]. These extraordinary attributes, including excellent aqueous processability, tunable surface functionalization, and high surface area, position GO and its derivatives as arguably the most promising candidates for optimized biomolecular interfacing in electrochemical biosensing [15–21].

The demand for such robust and versatile platforms is driven by the increasing sophistication of modern cell-based disease models [22–25]. Conventional diagnostic and research assays, including fluorescence imaging, polymerase chain reaction (PCR), and western blotting, provide reliable molecular data but are fundamentally limited by destructive sample preparation, endpoint readouts, and an inability to continuously monitor transient biological responses [26–31]. These limitations become particularly critical in advanced biological systems, such as three-dimensional (3D) spheroids and organoids [32–37], where dynamic temporal responses, ranging from metabolic shifts to neurotransmitter releases, occur over intervals of minutes to days [38–40]. Electrochemical biosensors offer a markedly advantageous solution by converting biochemical events into immediate electrical signals with high sensitivity and low sample consumption [41–45]. However, achieving reliable, real-time sensing in these volumetrically complex environments requires overcoming the inherent trade-offs between sensitivity and selectivity [46–48].

**Fig. 1:**
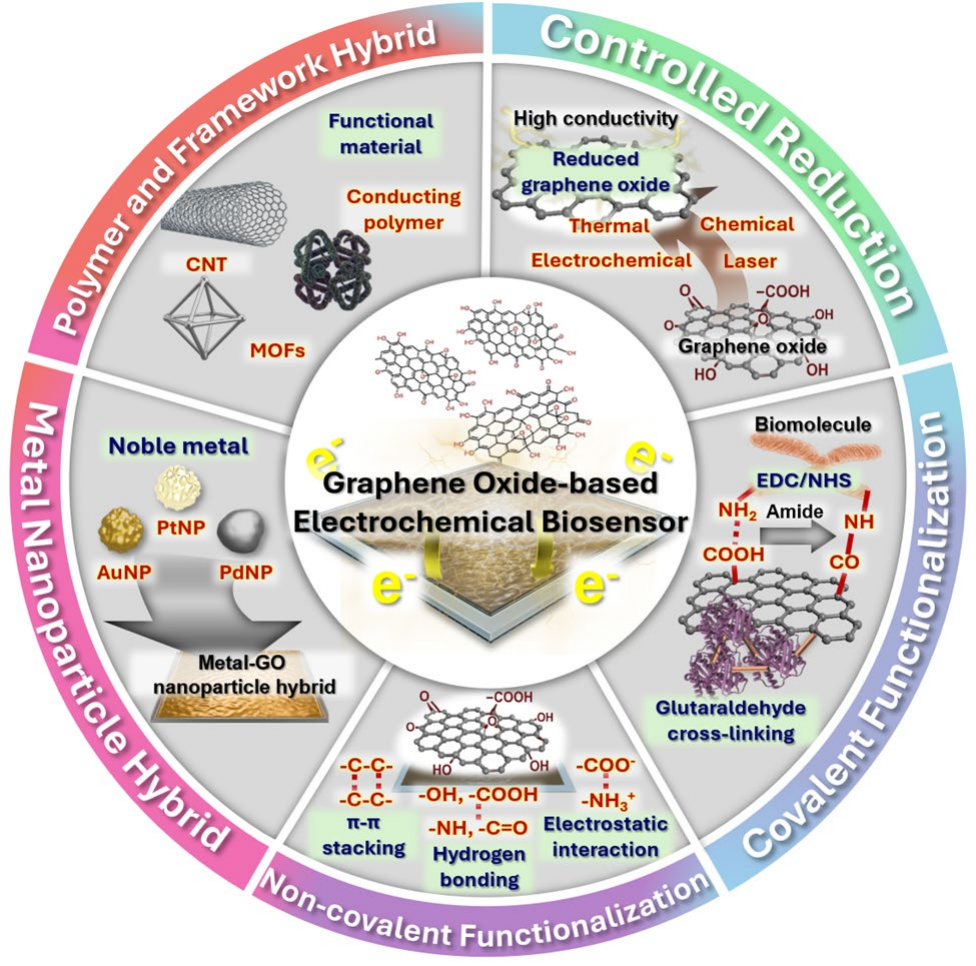
Schematic overview of the five graphene oxide functionalization strategies for electrochemical biosensing in biomedical applications discussed in this review

In this Review, we provide a rigorous and systematic examination of the diverse functionalization modalities employed to tailor GO-based interfaces for advanced bioanalytical applications. We delineate five primary strategic pillars that define the current state of the art in GO engineering: (1) controlled reduction techniques to precisely tune electrical conductivity and surface defect densities [49–51]; (2) covalent functionalization strategies, centered on amide bond formation and complemented by imine-based cross-linking, to ensure long-term interfacial stability [52–54]; (3) non-covalent modification via π–π stacking and electrostatic interactions to preserve the native conformation of sensitive bioreceptors [55–57]; (4) the integration of noble metal nanoparticles to achieve striking synergistic effects in electrocatalysis [58–60]; and (5) the construction of hierarchical architectures using conductive polymers and porous frameworks to significantly extend the analytical surface area [61–64] (Fig. 1).

By evaluating these methodologies, we demonstrate how tailored GO platforms facilitate the detection of a broad spectrum of analytes, ranging from small-molecule metabolites and neurotransmitters to nucleic acids and whole pathogens. Furthermore, while numerous excellent reviews already cover GO synthesis and general bioapplications, the integration of these engineered interfaces for real-time monitoring at complex biological interfaces, such as organoid-on-a-chip systems, remains an up-and-coming area that has yet to be comprehensively reviewed [65–67]. Finally, we address critical bottlenecks, including material standardization and biofouling, and outline a strategic roadmap for the transition of GO-based biosensors from proof-of-concept demonstrations into next-generation, clinically deployable diagnostic technologies.

## Controlled reduction of GO for tailored electrochemical performance

A key approach to improving the electrochemical performance of GO involves controlling the degree of oxidation. Electrical conductivity of the GO network depends directly on the connectivity of its sp²-hybridized carbon domains, which are determined by the density and distribution of oxygen-containing functional groups. Highly oxidized GO typically behaves as an insulator, while carefully controlled reduction methods gradually restore the sp² network and tune the electrical conductivity from insulating to semiconducting and conducting regimes depending on the degree of oxygen removal [68]. This tunability allows GO-based electrodes to be customized to meet specific electrocatalytic requirements of biosensing applications. However, conductivity recovery is not the only design parameter. Reduction also changes hydrophilicity, surface charge, defect density, roughness, biomolecule binding capacity, and film adhesion. Therefore, the optimal reduction condition for a biosensor is rarely the condition that maximizes conductivity alone. Rather, the optimal oxidation state is determined by the balance between electron transfer, analyte accessibility, interfacial stability, and functional group availability. The structural and electrical properties of GO depend on the selected reduction method, directly affecting the performance of the resulting biosensor. Thermal reduction restores the sp²-conjugated carbon network of GO by eliminating oxygenated groups, including carboxyl, hydroxyl, and epoxy functionalities [69–71]. Conventional high-temperature protocols operating above 300 °C can improve conductivity but frequently introduce irreversible structural defects, including lattice distortion, partial folding, wrinkled voids, and local aggregation. These changes can compromise film uniformity, electrode-to-electrode reproducibility, and long-term signal stability [72, 73].

**Fig. 2:**
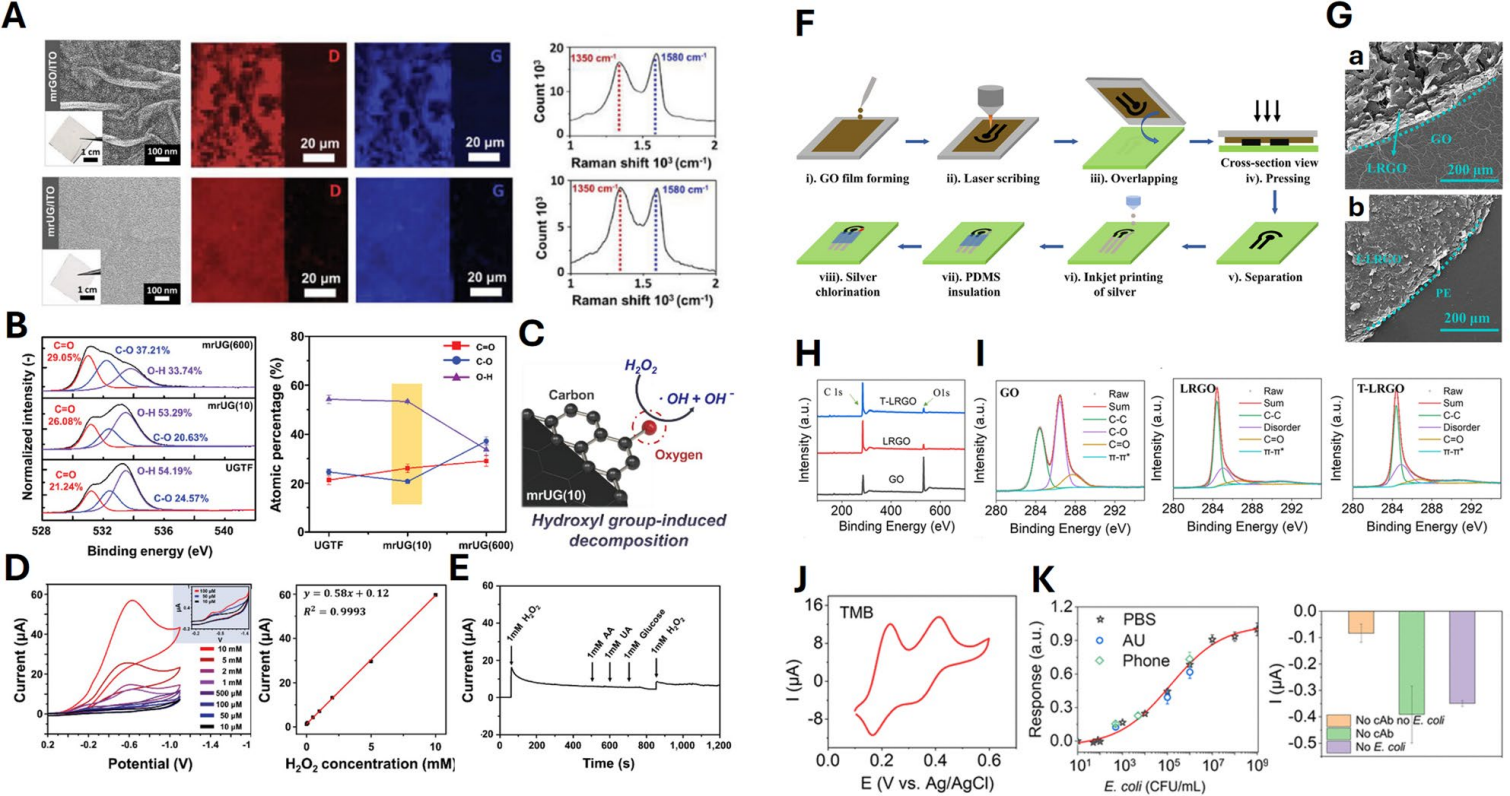
Controlled reduction strategies for tuning the oxidation state of GO. **A** FE-SEM and Raman D/G mapping images of mrGO/ITO and mrUG/ITO. **B** O 1s XPS spectra and atomic ratios of UGTF, mrUG(10), and mrUG(600) with a schematic of hydroxyl-induced H₂O₂ decomposition. **C** A schematic illustration showing mrUG based H₂O₂ sensing mechanism. **D** Cyclic voltammetric response of mrUG(10)/ITO toward H₂O₂, the corresponding calibration plot, **E** and amperometric selectivity test. Reproduced with permission from [1], Copyright 2023, Wiley-VCH GmbH. **F** Schematic of the laser scribing process and **G** SEM images of LRGO and T-LRGO. **H** XPS survey and **I** C 1s spectra of GO, LRGO, and T-LRGO. **J** Cyclic voltammetric response toward TMB, **K** calibration plot of the LRGO-based *E. coli* immunosensor, and selectivity verification. Reproduced with permission from [79], under CC-BY 4.0

Kim et al. [19] established the structural foundation for controlled GO reduction by fabricating an extremely uniform graphene oxide thin film (UGTF) on an indium tin oxide substrate through a three-step process solvent composition optimization at a water-to-ethanol ratio of 3:7, substrate preheating at 70 °C to suppress the coffee-ring effect [74, 75], and low-oxygen-concentration low-electrical-energy plasma treatment to fragment and smooth the deposited film, yielding a thickness of approximately 5.1 nm with a height variation of only 0.6 nm. Building on this platform, the same group of Kim et al. [1] subsequently demonstrated that the extreme thinness and uniformity of UGTF enabled rapid and homogeneous deoxygenation under mild conditions that left standard GO films incompletely or non-uniformly reduced [76]. UGTF was subjected to mild thermal annealing at 300 °C for 10 s to produce a hydroxyl-group-rich reduced film designated mrUG(10) (Fig. 2A). During this mild thermal annealing interval, carboxyl and epoxy groups were selectively eliminated while hydroxyl groups at flake edge structures were retained and partially rearranged into phenolic configurations, yielding an atomic percentage of O-H bonds of 53.3% in mrUG(10) compared to 33.7% in mrUG(600) in Fig. 2B, with a correspondingly low G/D ratio standard deviation confirming the suppression of partial folding and structural defects relative to conventionally reduced GO films. This hydroxyl-group-enriched architecture established a direct mechanistic link to electrochemical biosensing. The phenolic hydroxyl groups of mrUG(10) mediated hydrogen peroxide (H₂O₂) decomposition through surface-associated redox processes, generating reactive intermediates at the electrode surface and enabling metal-free electrocatalytic detection without requiring noble metal nanoparticles (Fig. 2C). Nevertheless, the mechanistic interpretation of metal-free H₂O₂ electrocatalysis on reduced GO should be treated carefully. Depending on reduction state, defect density, and oxygen functional group distribution, the dominant process may involve electron-transfer facilitation, adsorption-assisted decomposition, radical-associated pathways, or a combination of these mechanisms. Thus, phenolic hydroxyl groups are best understood as redox-active interfacial sites that can participate in H₂O₂ oxidation or reduction rather than as universally dominant radical-generating centers. As shown in Fig. 2D-E, the resulting platform achieved a linear range of 10 µM to 10 mM with a limit of detection of 6.21 µM, with signal stability confirmed across 20 repetitive detections in human plasma without measurable interference from ascorbic acid, uric acid, or glucose. This example illustrates how precise control of film morphology and reduction state can produce a metal-free sensing interface with reproducible electrochemical behavior.

While thermal annealing achieves GO reduction through sustained heat exposure, laser scribing offers a distinct physical route in which photothermal conversion of laser energy instantaneously raises the local surface temperature above 1,000 °C [77]. This rapid local heating removes oxygen-containing functional groups and restores the sp²-conjugated carbon network in a single maskless, chemical-free step that can simultaneously pattern electrode geometry without photolithography or clean room facilities [78]. This combination of spatial precision, direct writing, and scalable fabrication distinguishes laser reduction from thermal approaches. However, laser processing can also create local gradients in reduction degree, porosity, and defect density depending on laser power, scan speed, wavelength, substrate thermal conductivity, and GO film thickness. These parameters must be controlled to avoid excessive ablation or non-uniform electrode response. Zhao et al. [79] demonstrated its biosensing utility by applying CO₂ laser scribing to simultaneously reduce and pattern a drop-cast GO film on a polyester substrate, yielding laser-reduced graphene oxide (LRGO) electrodes that were subsequently stamp-transferred onto flexible substrates (Fig. 2F-G). X-ray photoelectron spectroscopy (XPS) analysis confirmed a C/O ratio increase from 2.7 in the original GO to 22.0 in LRGO [80], accompanied by a rise in sp² hybridized carbon content from 39.0% to 56.4%, verifying the effectiveness of the photothermal reduction and the restoration of graphene lattice conductivity (Fig. 2H-I). Critically, the reduction was not only surface-confined but penetrated uniformly through the bulk of the GO film, and a residual population of epoxide and hydroxyl groups was retained on the transferred electrode surface, providing anchoring sites for 1-pyrenebutanoic acid succinimide ester through π–π stacking [81], which in turn enabled covalent immobilization of polyclonal anti-*E. coli* antibodies for electrochemical immunosensing. The resulting platform detected pathogenic *E. coli* across a linear range of 917 to 2.1 × 10⁷ CFU/mL with a limit of detection of 283 CFU/mL using only 5 µL of sample, with selectivity confirmed against *Staphylococcus aureus* and *Salmonella typhimurium* and performance validated in spiked artificial urine (Fig. 2J-K). Beyond biosensing performance, the maskless, chemical-free fabrication process yielded more than 500 electrodes per day at a material cost below €0.12 per unit, establishing laser-scribed LRGO as a scalable and substrate-versatile platform readily adaptable to the detection of other pathogens by antibody substitution.

Electrochemical reduction offers a complementary route to thermal reduction by allowing precise [82], stepwise control over the state of oxygen functional group in GO through the applied potential and reduction cycles [83–85]. Because it can be performed at room temperature and directly on electrode surfaces, electrochemical reduction is especially attractive for fabricating rGO films on temperature-sensitive substrates [86]. Unlike thermal or chemical reduction, electrochemical reduction provides in situ control and can be coupled with simultaneous deposition, doping, or molecular assembly. However, electrochemical reduction is strongly influenced by electrolyte composition, pH, scan rate, potential window, substrate conductivity, film thickness, and interfacial adhesion. As a result, the same number of reduction cycles may not produce identical material states across different electrode configurations. Zhang et al. [87] reported a lysine-assisted electrochemical reduction strategy in which cyclic voltammetry (CV) was applied to achieve controlled deoxygenation of GO, with L-lysine simultaneously incorporated as a molecular crosslinker whose amine groups reacted with the epoxy and carboxyl groups of GO to form covalent inter-sheet bridges [88, 89]. These crosslinks directed the assembly of an electrochemically reduced graphene oxide-L-lysine (ERGO-Lys) composite with a crumpled, folded, and hierarchically structured 3D network, in which the folded junctions also served as sites of localized nitrogen incorporation derived from the lysine backbone, yielding a platform with an expanded electrochemically active surface area and a favorable microenvironment for subsequent biorecognition element attachment [90]. The ERGO-Lys platform demonstrated robust electrocatalytic glucose sensing with a limit of detection of 2.0 µM. In another study, Jarić et al. [91] further demonstrated that the number of CV cycles constitutes a critical design parameter, identifying 20 cycles as the optimal condition for converting a GO thin film into ERGO on a gold electrode surface [92], at which point the residual carboxyl and hydroxyl groups of ERGO provided anchoring sites for the subsequent immobilization of a thiol-modified MMP-2-specific aptamer via Au-S bond formation [93]. This ERGO-based aptasensor achieved a linear range of 10 pg/mL to 10 ng/mL with a limit of detection of 3.32 pg/mL and retained its selectivity in spiked human plasma samples, establishing electrochemical reduction as a versatile route to both composite electrode architectures and surface-functionalized aptasensing platforms.

Chemical reductants used in the reduction and functionalization of GO provide a versatile approach performed under mild conditions, though the choice of reducing agent involves specific trade-offs. Hydrazine (N₂H₄) represents one of the most established reducing agents for its deoxygenation efficiency and conductivity recovery [94, 95]. However, its toxicity and the incorporation of nitrogen heteroatoms (N-doping) limit its use in biological applications [96]. Sodium borohydride (NaBH₄) provides an alternative reduction pathway, but hydrogen gas produced during the reaction can disrupt the structural integrity of pre-assembled GO thin films [97–99]. Sulfuric acid (H₂SO₄) induces acid-catalysed dehydration, removing hydroxyl and epoxide groups [100]. Although H₂SO₄ restores the sp² carbon network without introducing metallic impurities, its corrosivity and the risk of surface sulfonation require careful post-treatment [101]. In contrast, mild-reducing agents such as potassium hydroxide (KOH) and L-ascorbic acid exhibit lower reduction efficiency but reduce the risk of structural degradation and chemical contamination [102–107]. KOH enables alkaline-driven deoxygenation without introducing toxic heteroatoms, providing a safer alternative to strong chemical agents despite its lower reduction efficiency. Similarly, L-ascorbic acid provides an environmentally green and biocompatible pathway. Although its reduction rate is slower, it avoids toxic residue accumulation and maintains the structural integrity of the films. Beyond their use as standalone reduction routes, chemical reductants are also frequently combined with thermal, laser, or electrochemical processes to fine-tune the oxidation state of GO and tailor the resulting electrochemical performance to the specific demands of each biosensing platform. Future controlled-reduction studies should report not only C/O ratio and Raman ID/IG values but also sheet resistance, electrochemical impedance, surface charge, wetting properties, film adhesion, and batch-to-batch variation. Such standardized characterization is essential because two GO films with similar elemental compositions may display different sensing performance if their flake size, defect topology, and interfacial connectivity differ.

## Covalent functionalization strategies for biorecognition

The abundant oxygen-containing functional groups on the GO surface serve as reactive sites for the covalent immobilization of diverse biomolecules. In particular, the carboxyl groups distributed on the basal plane and edges of GO provide suitable sites for selective coupling with amine-functionalized proteins, antibodies, aptamers, and enzymes, thereby forming stable bioconjugates [54, 108–110]. Such covalent functionalization has been extensively employed in GO-based biosensors because it enhances interfacial stability, improves detection reliability, and supports long-term monitoring of biomolecular interactions and biomarker responses [111–114]. However, covalent immobilization can also reduce biomolecule activity if active sites are sterically hindered or random orientation limits target accessibility. Therefore, covalent strategies require optimization of linker length, surface density, pH, activation time, blocking agents, and biomolecule orientation.

Among covalent routes, carbodiimide chemistry using 1-ethyl-3-(3-dimethylaminopropyl)carbodiimide (EDC) and N-hydroxysuccinimide (NHS) coupling is particularly common because it enables amide bond formation between carboxyl groups on GO and primary amines on biomolecules. This chemistry is attractive because it proceeds under aqueous conditions and does not require pre-functionalized pyrene linkers or metallic binding motifs. Nevertheless, EDC/NHS coupling is sensitive to pH and hydrolysis kinetics. EDC activates carboxyl groups to form O-acylisourea intermediates, which are unstable in aqueous solution; NHS or sulfo-NHS stabilizes the intermediate, but the activated ester can still hydrolyze before amine coupling occurs. Therefore, coupling efficiency depends strongly on reaction time, buffer composition, ionic strength, biomolecule concentration, and the accessibility of carboxyl groups. These factors are rarely standardized across GO biosensor studies, which contributes to differences in immobilization density and sensor reproducibility.

**Fig. 3:**
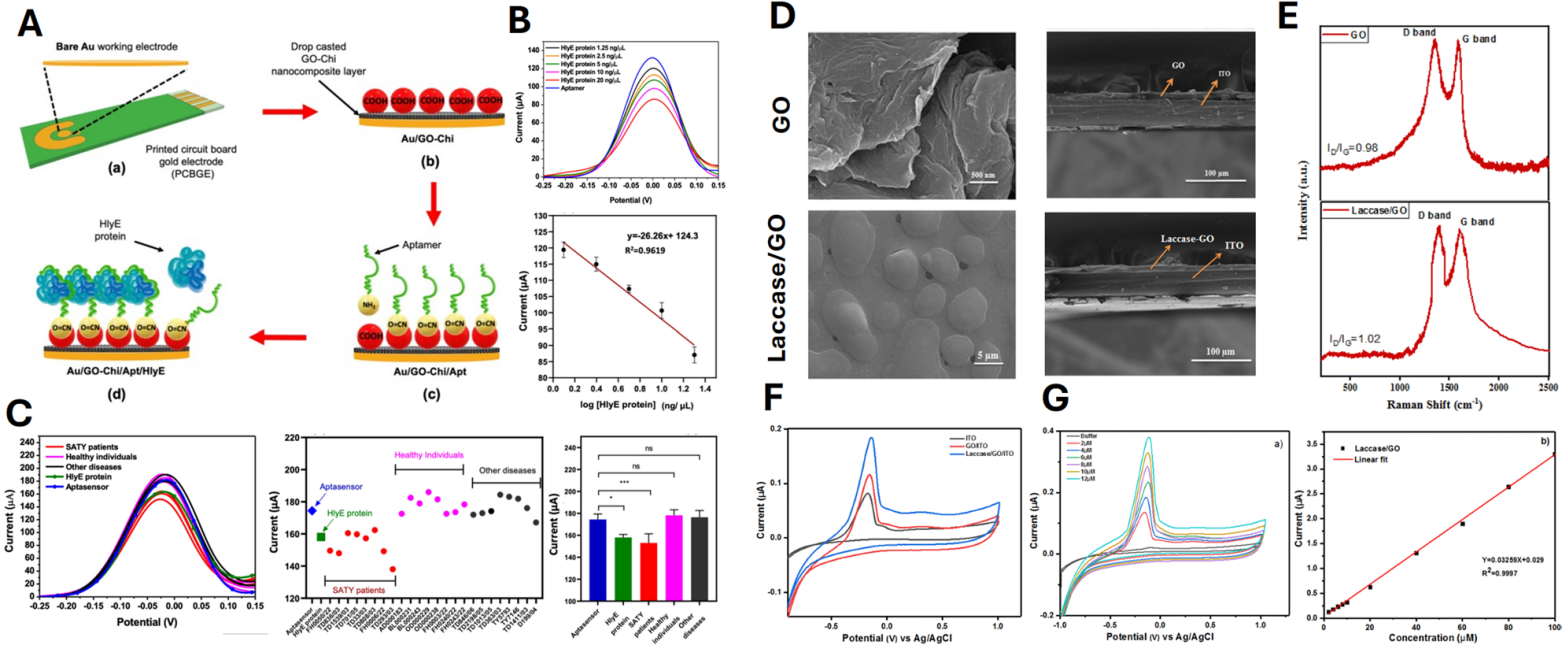
**A** Schematic illustration of the GO-Chi/aptamer biosensor fabrication for *Hemolysin E* (*HlyE*) detection. **B** DPV responses at increasing *HlyE* concentrations and corresponding linear calibration curve. **C** Selectivity validation in patient serum samples. Reproduced with permission from [116], Copyright 2024, Royal Society of Chemistry. **D** FE-SEM images of GO and laccase/GO surfaces. **E** Raman spectra confirming laccase immobilization. **F** Cyclic voltammetric response of ITO, GO/ITO, Laccase/GO/ITO and **G** linear calibration curve for GSH detection. Reproduced with permission from [114], Copyright 2025, Royal Society of Chemistry

The immobilization of biocompatible materials such as deoxyribonucleic acid (DNA) and ribonucleic acid (RNA) oligonucleotides has gained considerable research interest owing to their benefits for sensitive and selective detection of analytical target molecules [115]. Aptamers, consisting of single-stranded oligonucleotides, represent a versatile alternative to antibody-based detection methods. These biorecognition elements possess several distinct features including small size, facile synthesis, robust stability, and flexible modification capability, rendering them suitable for a range of biosensing applications compared with conventional approaches. Awang et al. [116] developed an aptasensor that utilized surface modification of GO for aptamer immobilization on a gold electrode to detect *Hemolysin E* (*HlyE*), a virulence factor of *Salmonella* Typhi (Fig. 3A). The gold electrode was coated with a GO/chitosan nanocomposite, and the carboxyl groups on the GO surface were activated through EDC/NHS coupling chemistry to covalently anchor the terminal amino groups of aptamers via amide linkages. Chitosan contributed film-forming ability, amine-rich chemistry, and improved biocompatibility, while GO provided high surface area and carboxyl-rich anchoring sites. Quantification was achieved by monitoring the differential pulse voltammetric (DPV) response of the [Fe(CN)₆]³⁻/⁴⁻ redox probe, in which Apt-*HlyE* binding suppressed interfacial electron transfer and produced a concentration-dependent decrease in DPV peak current at the GO surface. Strictly speaking, this platform is label-free with respect to the target recognition event but not fully reagent-free because it relies on an external redox probe for transduction. The aptasensor exhibited a linear response over 1.25 to 20 ng/µL with a limit of detection of 0.137 ng/µL for *HlyE* detection (Fig. 3B). Furthermore, the selectivity and sensitivity were validated through serum analysis from infected patients, healthy individuals, and other disease patient groups. The patient group samples exhibited an average current peak of 153.09 ± 8.3 µA, showing a distinct current variation compared to the control group peak (174.40 ± 5.0 µA), successfully achieving detection differentiation (Fig. 3C). These results establish EDC/NHS-mediated covalent immobilization on GO as a viable strategy for sensitive electrochemical detection of pathogen-associated virulence proteins relevant to early-stage clinical diagnosis.

Beyond aptamer-based recognition, GO surface chemistry is equally amenable to enzyme immobilization, in which glutaraldehyde cross-linking provides an alternative covalent route that produces stable enzyme films suitable for small-molecule biosensing. Glutaraldehyde reacts primarily with amine groups in enzymes or matrix proteins, producing imine or related cross-linked structures that stabilize enzyme layers. This amine-aldehyde condensation is a Schiff-base reaction, and the equivalent imine linkage can also be formed directly on aldehyde-modified GO, in which aldehyde groups introduced by controlled oxidation condense with the primary amines of antibodies, aptamers, or enzymes to anchor them covalently. However, excessive cross-linking can reduce enzyme flexibility, restrict substrate diffusion, and decrease catalytic activity. Thus, glutaraldehyde concentration and reaction duration must be balanced to preserve enzyme function while preventing leaching. Ukirade et al. [114] developed a laccase-based biosensor for the detection of glutathione (GSH), an intracellular antioxidant whose depletion reflects oxidative stress in living cells. The platform was constructed by drop-casting GO onto an indium tin oxide (ITO) electrode, after which a laccase and gelatin mixture was spread over the GO surface and cross-linked by 2.5% glutaraldehyde, forming a stable enzyme film anchored to the GO interface. The oxygen-containing functional groups of GO supported uniform laccase distribution, while the gelatin matrix preserved enzymatic activity through the cross-linked network (Fig. 3D). Field-emission scanning electron microscopy (FE-SEM) analysis confirmed the transformation from a wrinkled GO surface into a compact film with uniformly distributed spherical laccase, and in Fig. 3E, Raman spectra showed an increase in ID/IG ratio from 0.98 to 1.02 upon laccase incorporation, verifying successful enzyme integration. Detection of GSH proceeded through enzymatic oxidation of GSH to oxidized glutathione (GSSG) by laccase, followed by electrochemical regeneration of GSSG at the working electrode, with the resulting anodic peak current measured by CV (Fig. 3F). Compared with bare ITO and GO/ITO electrodes, the laccase/GO/ITO electrode delivered a 2.25-fold enhancement in oxidation peak current toward 4 µM GSH, attributed to the cooperative contribution of GO surface area and laccase catalytic activity. The biosensor exhibited a linear response over 1 to 100 µM with a limit of detection of 0.89 µM and a sensitivity of 6.51 µA/µM (Fig. 3G). The platform retained over 95% of its initial response after 30 days of storage at 4 °C and yielded recovery values between 95 and 105% in human blood samples, indicating that glutaraldehyde-mediated covalent enzyme immobilization on GO provides a stable interface for clinically relevant electrochemical detection of small antioxidant molecules.

Overall, covalent GO functionalization provides robust interfaces for biosensors that must operate in complex media or undergo repeated washing. Its main advantages are chemical stability, controlled molecular anchoring, and compatibility with antibodies, aptamers, enzymes, peptides, and polymer matrices. Its main limitations are potential loss of biomolecule activity, random orientation, reduced surface regenerability, and sensitivity to coupling conditions. Future covalent strategies should incorporate oriented immobilization motifs, antifouling layers, and quantitative surface characterization to link immobilization density with analytical performance.

## Non-covalent functionalization strategies for biorecognition

In contrast to covalent immobilization, which depends on irreversible chemical linkages [117, 118], non-covalent functionalization provides a complementary strategy for GO-based biosensing that exploits reversible interfacial interactions while preserving biomolecule activity [119]. The heterogeneous surface of GO, featuring both sp² carbon domains and oxygen-containing functional groups, supports three principal modes of non-covalent association, including π–π stacking with aromatic analytes, hydrogen bonding via hydroxyl and carboxyl groups, and electrostatic interactions mediated by the negatively charged carboxylate species under physiological pH [120]. This versatility allows fine-tuning of molecular recognition through controlled surface chemistry and environmental conditions, rendering non-covalent platforms suitable for regenerable sensors and real-time monitoring applications. However, non-covalent interactions are intrinsically sensitive to pH, ionic strength, temperature, serum proteins, and competing biomolecules. For example, electrostatic attractions between GO and positively charged polymers can be screened in high-salt physiological media, while adsorbed proteins can be displaced by proteins with stronger surface affinity. π–π stacking is particularly effective for aromatic small molecules, nucleobases, and pyrene-functionalized linkers, but it should not be generalized as a universal protein immobilization mechanism. For globular proteins, adsorption depends on hydrophobic patches, aromatic amino acid residues, charge distribution, and protein conformation. These interactions translate into measurable turnover at the GO surface. Nonspecific protein adsorption from serum begins within the first five minutes of exposure on unprotected carbon-based electrodes, progressively degrading the baseline signal. Under continuous flow on graphene field-effect transistors, surface-bound aptamer-protein complexes dissociate with off-rates of approximately 0.1 to 1 s⁻¹, and these rates increase with ionic strength and temperature [121]. The strength of physisorption is likewise tunable, as single-stranded DNA adsorbs more rapidly and binds more tightly on GO for shorter sequences and at higher ionic strength, which in turn governs the rate of probe release during target detection [122]. Therefore, non-covalent GO biosensors require careful evaluation of desorption, fouling, and long-term interfacial stability. The following subsections examine three interaction modes in turn, illustrating how each mechanism translates into distinct biosensing performance.

**Fig. 4:**
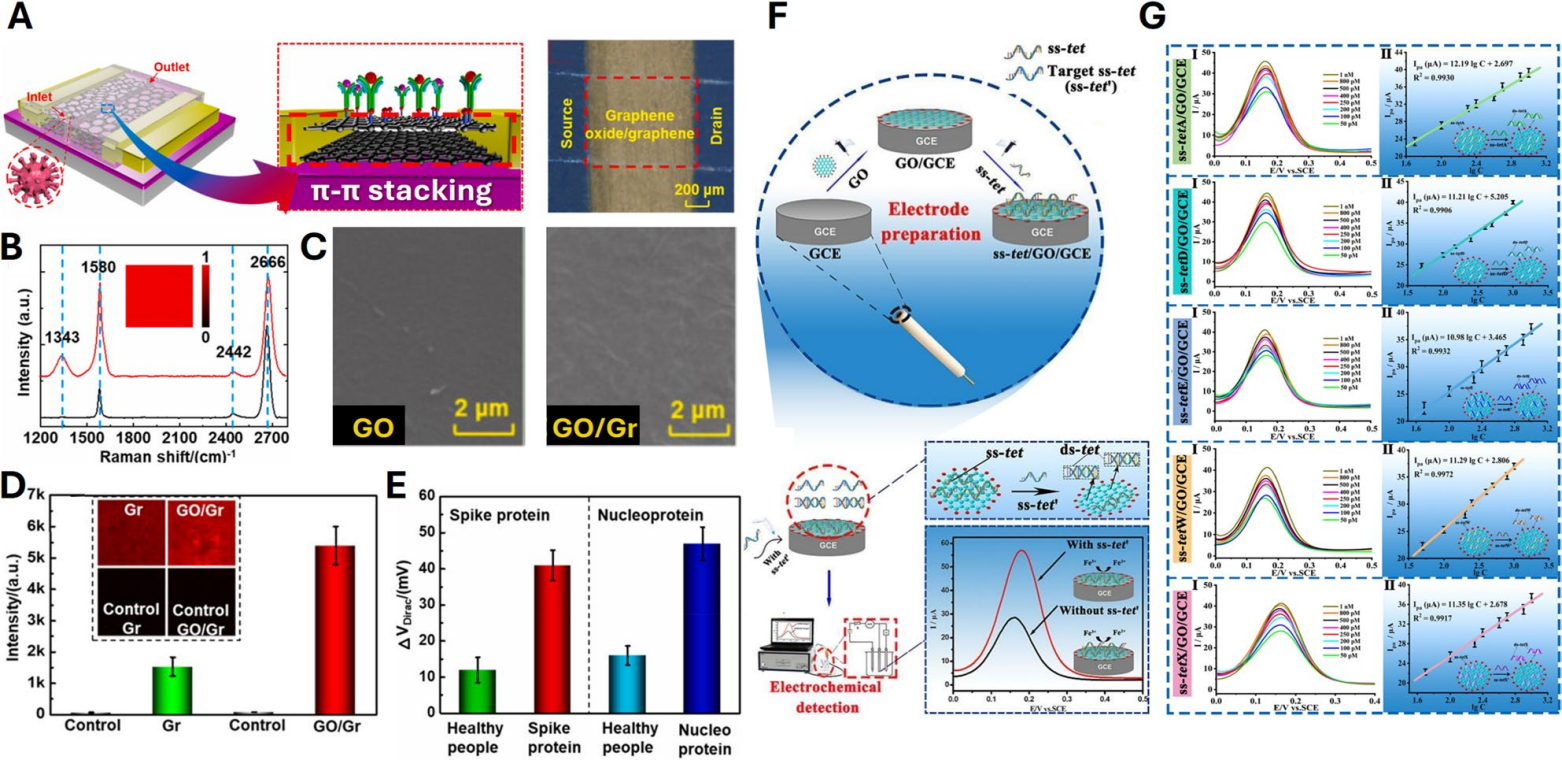
**A** Schematic illustration of the GO/graphene FET biosensor for SARS-CoV-2 detection through π–π stacking. **B** Raman spectra and mapping of GO/Gr heterostructure. **C** Optical images of GO and GO/Gr surfaces. **D** Antibody immobilization density on Gr and GO/Gr surfaces. **E** Dirac voltage shifts for spike protein and nucleoprotein detection. Reproduced with permission from [123], Copyright 2022, Elsevier. **F** Schematic of ss-tet/GO electrode preparation and *tet* gene detection mechanism. **G** DPV responses and linear calibration curves for five tet subtypes. Reproduced with permission from [127], Copyright 2024, Elsevier

Among the three non-covalent modes, π–π stacking serves as the dominant driving force when aromatic analytes or graphitic surfaces are involved. Gao et al. [123] exploited this principle to construct a GO-based field-effect transistor (FET) biosensor for SARS-CoV-2 protein detection [124, 125] (Fig. 4A), in which GO nanosheets self-assembled onto monolayer graphene through π–π stacking between the sp² basal planes of the two materials, forming a GO/graphene heterostructure within a microfluidic channel at room temperature without chemical modification (Fig. 4B-C). Capture antibodies were subsequently anchored onto the GO overlayer through cooperative π–π stacking and hydrogen bonding with the hydroxyl and carboxyl groups of GO, raising the antibody immobilization density by 3.6-fold compared with the bare graphene channel (Fig. 4D). The denser antibody loading translated directly into improved electrical transduction, with the biosensor exhibiting a linear response over 10 fg/mL to 100 pg/mL spike protein and a limit of detection of approximately 8 fg/mL, representing a threefold sensitivity enhancement over the unmodified graphene FET (Fig. 4E). The platform recovered spiked SARS-CoV-2 proteins from human throat swab buffer within 20 min while maintaining selectivity against AFP and nucleoprotein interferents, establishing π–π-driven GO assembly as a chemical-free route to amplify biorecognition density on graphitic biosensing interfaces.

Beyond π–π stacking, the deprotonation of edge-localized carboxyl groups imparts a negative surface charge to GO at physiological pH, thereby enabling electrostatic self-assembly with positively charged molecular species without covalent crosslinking. Monkrathok et al. [126] applied this principle to construct an NAD-dependent glucose dehydrogenase (NAD-GDH) biosensor, in which nanocolloidal GO was first drop-cast onto a screen-printed carbon electrode, followed by a mixture of GDH, ferrocene-modified linear poly(ethylenimine) (LPEI-Fc), and the EGDE crosslinker. The Coulombic attraction between the negatively charged GO surface and the protonated amine backbone of LPEI-Fc anchored the redox polymer onto GO, while complementary π-stacking between GO and the underlying carbon electrode produced a uniformly distributed enzyme/polymer film verified by FE-SEM. Within this architecture, GDH catalyzed glucose oxidation with concomitant reduction of NAD⁺ to NADH, and the pendant ferrocene groups along the LPEI backbone shuttled electrons from the enzymatic reaction to the electrode through self-exchange collisions, supporting NADH oxidation at an applied potential of 0.35 V. The GO underlayer reduced the charge-transfer resistance of the bare electrode from 1745 Ω to 191 Ω, and the integrated flow injection biosensor exhibited a linear amperometric response over 1.0 to 40 mM glucose with a limit of detection of 0.28 mM, covering the physiological glucose range of 2.8 to 22.2 mM. The platform was further validated by quantifying glucose in a commercial sports drink in agreement with a commercial glucometer, demonstrating that electrostatic GO/redox-polymer self-assembly delivers a stable mediated electron-transfer interface for clinically relevant glucose monitoring.

Beyond electrostatic interactions, the hydroxyl and carboxyl groups distributed across the basal plane of GO contribute hydrogen-bonding sites that cooperate with π-stacking when biomolecules carrying both aromatic rings and exposed donor or acceptor groups interact with the GO surface. Nucleic acids are particularly suitable for such dual-mode interaction because their nucleobases can interact with graphitic domains while phosphate backbones and nucleobase functional groups participate in hydrogen bonding and electrostatic interactions. Xing et al. [127] applied this dual-mode interaction to construct an electrochemical sensor for tetracycline resistance genes (*tet*) in environmental water, in which 20-mer single-stranded probe DNA (ss-tet) was non-covalently adsorbed onto a GO-modified glassy carbon electrode through π–π stacking between the aromatic nucleobases and the sp² basal plane of GO, accompanied by hydrogen bonding between nucleobase pairs (Fig. 4F). Density functional theory calculations confirmed favorable adsorption energies of -0.48 to -0.61 eV between GO and the four nucleobases, supporting the spontaneous physisorption of ss-tet onto GO without covalent crosslinking. Detection proceeded through hybridization with the complementary target sequence (ss-tet’) in PBS, which formed double-stranded ds-tet whose nucleobases were shielded inside the helical structure, weakening the affinity for GO and triggering release of the duplex from the electrode surface. The released ssDNA probe restored interfacial electron transfer of the [Fe(CN)₆]³⁻/⁴⁻ redox couple, producing a concentration-dependent increase in DPV peak current over a linear range of 50 pM to 1 nM with a limit of detection of 50 pM (Fig. 4G). The sensor was prepared in under 35 min, distinguished five *tet* subtypes with relative standard deviation (RSD) below 4.43%, and quantified *tetX* in real river and sewage samples in agreement with reverse transcription quantitative PCR (RT-qPCR), establishing non-covalent GO/DNA assembly as a label-free and rapid platform for environmental nucleic acid sensing.

While the functionalization strategies discussed above expand the electrochemical performance and biorecognition capability of GO-based platforms, single-material and single-strategy approaches remain constrained in their ability to simultaneously optimize sensitivity, selectivity, electrocatalytic activity, and long-term stability in complex biological environments. Covalent systems provide stability but may sacrifice activity or regenerability; non-covalent systems preserve biomolecule conformation but may suffer from leaching; and reduced GO improves conductivity but can lose functional group density. To address these concurrent demands, researchers have increasingly developed hybrid nanocomposite platforms that integrate GO or rGO with complementary nanomaterials.

## Metal nanoparticle hybrid composites for electrochemical sensing

Hybrid nanocomposites that combine GO or rGO with complementary nanomaterials have emerged as a key strategy to overcome the inherent limitations of single-material platforms and improve electrochemical biosensing performance [3, 128]. By integrating the large surface area and functionalization diversity of GO with the specialized properties of additional components, these composite systems generate synergistic effects that neither constituent can achieve independently, yielding improvements in sensitivity, selectivity, and long-term stability [129, 130]. The electrochemical performance gains achieved through this hybrid approach exceed those attainable through surface functionalization of GO alone [131]. Depending on the chemical nature of the incorporated material, these hybrid composites are broadly categorized into metal nanoparticle composites and polymer- and framework-based hybrid composites [132].

Metal nanoparticles provide sensitive electrocatalytic activity that promotes the redox reactions of biomolecules [133], but their practical deployment in biological sensing environments is critically constrained by self-aggregation. Elevated ionic strength and pH fluctuations in physiological media progressively reduce electrostatic repulsion between particles, accelerating coalescence and degrading both sensitivity and long-term signal reproducibility [134, 135]. Addressing this instability requires a support material that simultaneously enables uniform nanoparticle dispersion, efficient interfacial electron transfer, and stable biomolecular functionalization. GO fulfills each of these requirements through distinct and complementary mechanisms [136]. Its abundant oxygen-containing functional groups carboxyl, hydroxyl, and epoxy groups act as anchoring sites that coordinate metal ions during nanoparticle nucleation and stabilize the resulting particles against aggregation [133]. Upon partial reduction to rGO, the restored sp²-conjugated network lowers interfacial charge-transfer resistance and accelerates electrocatalytic kinetics. This complementary behavior between GO and metal nanoparticles yields composites whose electrochemical performance exceeds that of either constituent alone [137]. Gold, platinum, and palladium combine high electrocatalytic activity with the chemical stability and biocompatibility required for prolonged operation in physiological media, which accounts for their predominant use in GO-based cell and organoid biosensing. Silver and copper nanoparticles are also electrocatalytically active and have been hybridized with GO, but their application in biological environments is constrained by characteristic instabilities. Silver undergoes oxidative dissolution that releases Ag⁺ ions, introducing cytotoxicity toward cultured cells and progressive signal drift [138], whereas copper is prone to surface oxidation and dissolution in oxygenated, chloride-containing media, reducing reproducibility and biocompatibility [139]. The oxygen-containing groups of GO can coordinate and partially stabilize these metals, yet the residual ion leaching and redox instability limit their suitability for the long-term cell and organoid measurements emphasized here.

**Fig. 5:**
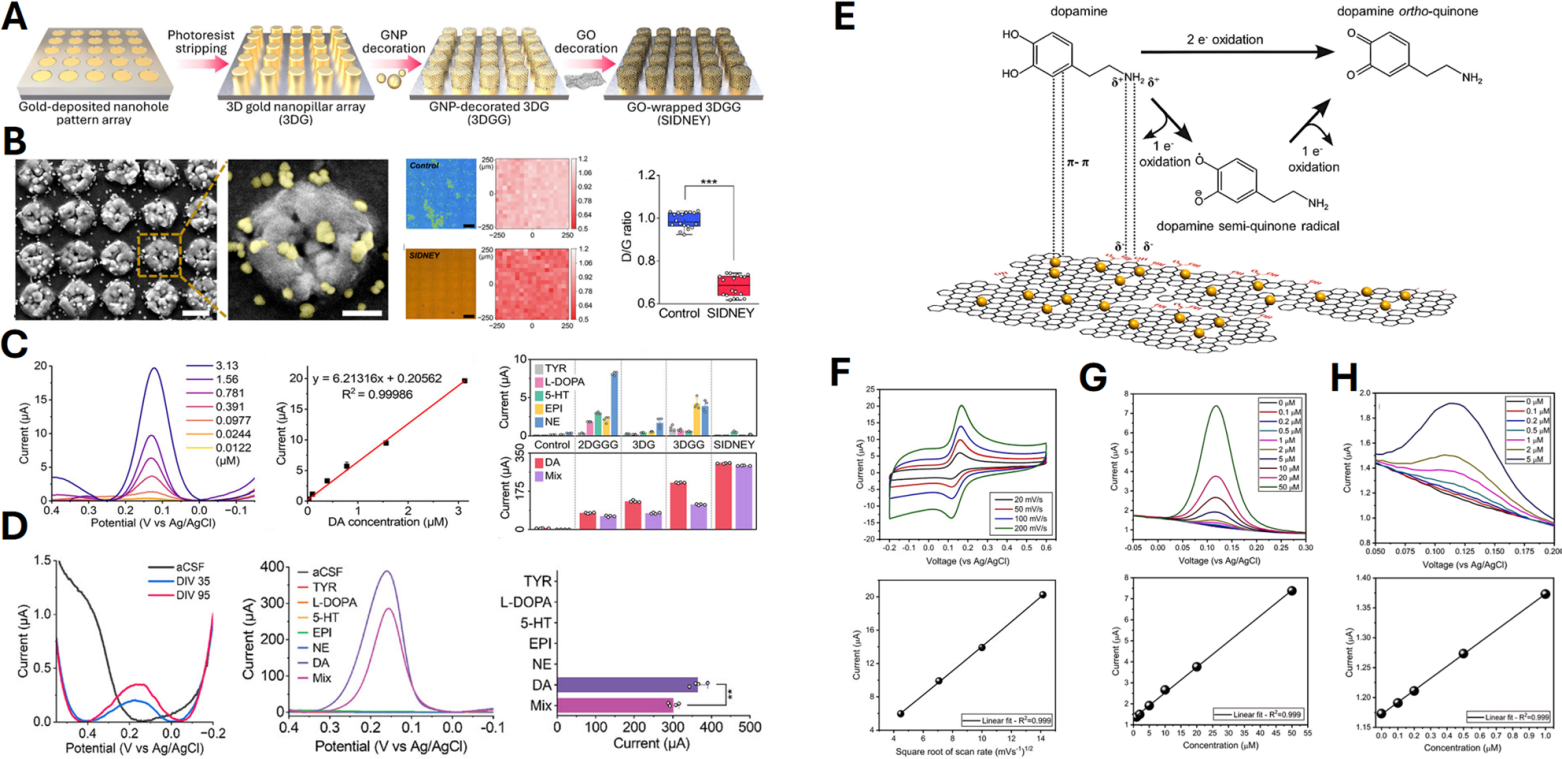
**A** Schematic of SIDNEY fabrication and characterization. **B** Schematic of DA oxidation mechanism on SIDNEY. **C** DPV responses to DA, linear calibration curve, and selectivity comparison against interferents. **D** DPV responses of midbrain organoids at DIV 35 and DIV 95 with selectivity validation. Reproduced with permission from [140], Copyright 2026, Wiley. **E** Schematic of DA oxidation mechanism on atomically dispersed Pd/GO. **F** Cyclic voltammograms at varying scan rates and corresponding linear plot of peak current versus square root of scan rate. **G** DPV responses at increasing DA concentrations and linear calibration curve. **H** DPV responses at low DA concentrations and linear calibration curve. Reproduced with permission from [150], Copyright 2025, Elsevier

Among noble metal nanoparticles, gold nanoparticles (AuNPs) have been most extensively integrated with GO for cell-based electrochemical biosensing. Kang et al. [140] developed SIDNEY (Smart Interfacial Dopamine-sensing platform for NEurons and organoid physiologY), a GO-wrapped hierarchical gold nanopillar hybrid for label-free, non-destructive, real-time electrochemical monitoring of dopamine (DA) under live-cell conditions (Fig. 5A). The platform consists of vertically aligned gold nanopillars as a 3D scaffold, onto which secondary AuNPs are decorated, and a thin GO layer is subsequently coated. In this architecture, each component serves a distinct and non-redundant role, where gold nanopillars expand the electrochemically active surface area and enhance electron transfer kinetics; AuNPs further increase electroactive surface area and amplify redox reactivity; and GO confers molecular selectivity and anti-fouling behavior through π–π interactions, hydrogen bonding, and electrostatic interactions, while simultaneously promoting robust cell adhesion on the electrode surface (Fig. 5B). SIDNEY achieved a limit of detection of 29.5 nM in phosphate-buffered saline and 7.51 nM in artificial cerebrospinal fluid, with high selectivity against structurally similar interferents including ascorbic acid, serotonin, and epinephrine (Fig. 5C). The platform further supported long-term culture and differentiation of both SH-SY5Y cells and iPSC-derived dopaminergic neurons directly on the electrode and enabled real-time functional assessment of dopaminergic activity in midbrain organoids at different developmental stages (35 and 95 days in vitro, DIV) without labeling or sample destruction (Fig. 5D). In this system, the GO-wrapped hierarchical gold nanopillar architecture provided sufficient electrochemical sensitivity to resolve dopaminergic activity within 3D tissue-mimicking constructs, demonstrating that the GO/AuNP hybrid interface can sustain real-time neurochemical monitoring from 2D neuronal cultures to volumetrically complex organoid systems.

Platinum nanoparticles (PtNPs) offer a complementary catalytic profile distinct from AuNPs, exhibiting particularly strong electrocatalytic activity toward H₂O₂ reduction and glucose oxidation [141], which makes rGO-PtNP composites well-suited for non-enzymatic detection of oxidative stress markers and metabolic substrates [142]. Zhu et al. [143] developed a multilayer biosensor architecture for in situ electrochemical monitoring of intracellular oxidative stress in 3D cancer cell models. A screen-printed carbon electrode was first modified with reduced graphene oxide through GO drop-casting and CV-driven reduction, after which platinum nanoparticles approximately 40 nm in diameter were electrolytically deposited onto the rGO surface from a K₂PtCl₆ precursor at scan potentials between − 1.5 V and 0 V. NCI-H1975 lung cancer cells were then encapsulated within a GelMA/GO hydrogel layer applied directly onto the PtNPs/rGO/SPCE, and ultraviolet-mediated photocrosslinking immobilized the cells while a second CV step reduced the GO within the hydrogel to rGO, establishing electron transfer between the encapsulated cells and the underlying electrode. In this dual-layer architecture, rGO served two distinct roles, with the lower layer providing a conductive scaffold for uniform Pt nanoparticle dispersion and electrocatalytic H₂O₂ reduction at -0.14 V, while the upper hydrogel-embedded rGO maintained electrical continuity between the 3D cellular microenvironment and the transducing surface. The platform achieved a linear range of 1 to 10 µM with a limit of detection of 0.65 µM and a sensitivity of 4.868 µA/µM for H₂O₂, with negligible interference from glucose, oxalic acid, and ascorbic acid, and was applied to evaluate the prooxidant capacity of honokiol, a Magnolia-derived anti-cancer compound, demonstrating a biphasic dose-response in which low concentrations promoted NCI-H1975 proliferation while concentrations above 35.97 µM upregulated oxidative stress and inhibited cancer cell viability. Beyond cell-based detection environments, the catalytic activity of PtNP/rGO composites can be further enhanced through bimetallic design strategies. Chen et al. [144] extended this design principle to a bimetallic architecture by depositing core-shell Au@Pt nanoparticles onto electrochemically reduced GO, where the residual oxygen-containing functional groups anchored the nanoparticles against displacement and the restored π-conjugated network facilitated rapid charge transport. The Au core suppressed Pt shell aggregation and promoted nanochannel formation at the Au-Pt interface that accelerated intramolecular charge transfer, reducing the interfacial charge-transfer resistance to 12 Ω and achieving a linear range of 1–8 mM with a limit of detection of 0.078 mM, a sensitivity of 28 µA·mM⁻¹·cm⁻², and 96% signal retention after 15 days. Li et al. [145] further advanced PtNP immobilization specificity by co-incorporating B, S, and N heteroatoms into GO via hydrothermal treatment, producing BSN-rGO in which nitrogen atoms formed Pt-N covalent bonds upon NaBH₄ reduction of H₂PtCl₆ that prevented PtNP detachment under operational conditions, while the modified band structure enhanced interfacial electron transfer kinetics. Applied to cardiac troponin I (cTnI) immunosensing, this platform achieved a linear range of 0.1 pg/mL to 50 ng/mL with a limit of detection of 0.082 pg/mL.

Palladium nanoparticles (PdNPs) extend this catalytic versatility by enabling H₂O₂ reduction at lower operating potentials than PtNPs [146, 147], reducing signal overlap with co-existing electroactive species in biological environments, while their high coordination affinity for N and S heteroatoms makes them particularly amenable to atomic-scale dispersion on GO supports [148]. Xi et al. [149] developed a self-supported freestanding electrode in which GO served as the starting material for a nitrogen- and sulfur-co-doped porous graphene film (NSPGF) through high-temperature annealing under an inert atmosphere. The substitutional N and S dopants created anchoring sites that coordinated Pd²⁺ ions through a spontaneous redox reaction, dispersing PdNPs uniformly without surfactants or binders and lowering the interfacial charge-transfer resistance from 214.2 Ω to 80.9 Ω. The platform distinguished normal human colon epithelial cells from colorectal cancer cells by quantifying extracellular H₂O₂ released per cell upon chemical stimulation, across a linear range of 0.5 µM to 2.0 mM with a limit of detection of 0.1 µM and a sensitivity of 665 µA·cm⁻²·mM⁻¹. Ozbakir et al. [150] pushed this concept further by reducing Pd loading to the atomic scale palladium(II) acetate was incorporated into GO via wet impregnation at room temperature, and subsequent inert-atmosphere annealing removed the organic ligands while retaining the residual carboxyl, hydroxyl, and epoxy groups that coordinated isolated Pd atoms in a -O-Pd-O- surface configuration (Fig. 5E). The diffusion-controlled nature of the DA oxidation was confirmed by a linear correlation between peak current and the square root of scan rate (Fig. 5F), and the platform achieved a linear range of 0.1–50 µM with a limit of detection of 55 nM (Fig. 5G-H), outperforming both Pd nanoparticle-loaded GO (128 nM) and pure GO (272 nM) under identical conditions.

Taken together, metal nanoparticle/GO composites demonstrate how GO-derived supports can transform nanoparticle catalysts from aggregation-prone colloidal materials into stable electrochemical interfaces. Nevertheless, these systems introduce important translational challenges. Noble metal loading increases cost, nanoparticle leaching must be evaluated under physiological flow and long-term culture conditions, and catalytic activity may be degraded by protein fouling or cell-secreted extracellular matrix. Therefore, future metal/GO hybrid biosensors should include rigorous stability testing under serum-containing media, repeated stimulation cycles, and extended operation at living interfaces.

## Polymer and framework hybrid composites

While the metal nanoparticle composites discussed above rely primarily on the electrocatalytic activity of the metal component, a complementary strategy integrates GO with conductive polymeric and framework materials including conducting polymers, carbon nanotubes, and porous metal-organic frameworks [62, 151] to exploit the structural organization capabilities that emerge at the GO/partner interface [152]. In these hybrid systems, the polymeric or framework partner does not merely provide electrical conductivity but actively governs the 3D architecture of the composite, determining analyte accessibility, surface area, and the spatial distribution of oxygen-containing functional groups [153]. The resulting structural diversity enables sensing capabilities from single-analyte detection at living cell interfaces to simultaneous multianalyte discrimination in biological fluids that are difficult to attain with metal nanoparticle-based platforms alone.

**Fig. 6:**
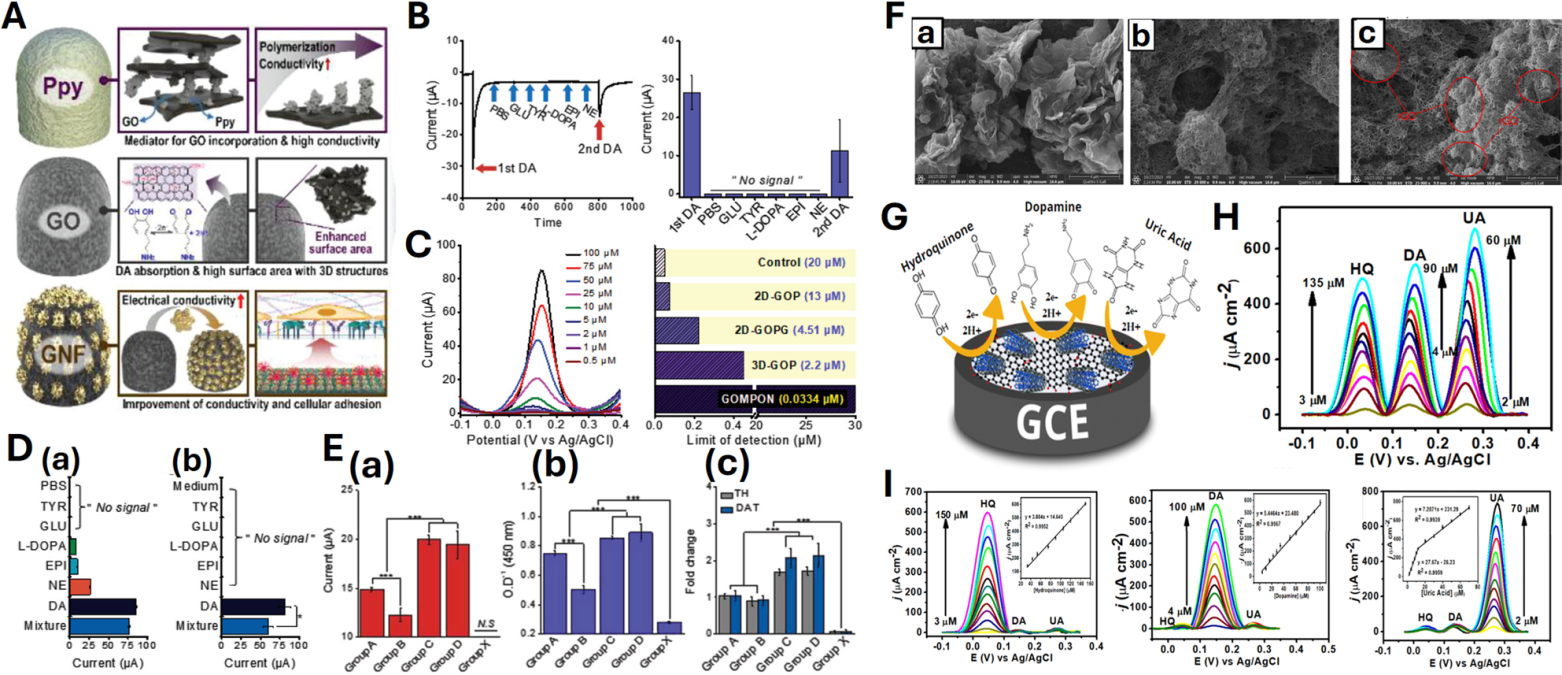
**A** Schematic of DA oxidation on GOMPON. **B** Chronoamperometric selectivity against structurally similar interferents. **C** DPV responses to DA and linear calibration curve. **D** Selective DA detection from cells cultivated on the electrode. **E** DPV monitoring of DA release from iPSC-derived dopaminergic neurons (groups A-D) versus unstimulated control X, with (Ea) DPV quantification, (Eb) ELISA, and (Ec) TH/DAT qPCR. Reproduced with permission from [154], Copyright 2023, Wiley. **F** Surface image of (Fa) rGO, (Fb) MWCNT and (Fc) rGO/MWCNT electrodes. **G** Schematic of DPV responses for simultaneous HQ, DA, and UA detection. **H**-**I** Linear calibration curves for the three analytes. Reproduced with permission from [155], Copyright 2024, Royal Society of Chemistry

Cho et al. [154] developed GOMPON (graphene oxide-incorporated metallic polymer nanopillar arrays), a 3D hybrid nanoarchitecture in which nanosized GO and pyrrole monomers were co-electropolymerized within lithographically defined nanohole arrays to form vertically aligned GO/polypyrrole (PPy) nanopillars (Fig. 6A). The nanosized dimensions of GO were essential, as only nGO could penetrate the 400–450 nm diameter nanoholes to participate in 3D pillar formation alongside PPy [62], whereas micro sized GO formed a blanket-like film that blocked analyte access to the electrode surface. Within the resulting architecture, GO served a dual chemical function, where its aromatic basal plane enabled π–π stacking interactions with the catechol ring of DA, while its residual hydroxyl and carboxyl groups facilitated hydrogen bonding collectively providing the molecular basis for selective DA recognition over structurally similar interferents including tyrosine, L-DOPA, epinephrine, and norepinephrine (Fig. 6B). PPy complemented this by contributing electrostatic attraction of the positively charged DA and expanding the 3D GO exposure in the vertical direction. Gold nanoclusters subsequently deposited onto the pillar tips amplified electrocatalytic signal transduction by 2.35-fold relative to the gold-free electrode. The platform achieved a linear detection range of 0.5–100 µM with a limit of detection of 33.4 nM (Fig. 6C) and enabled real-time, nondestructive electrochemical monitoring of DA release from cells cultivated directly on the electrode surface (Fig. 6D). DPV monitoring across the 12-day differentiation period showed progressively increasing DA release in induced dopaminergic neuron groups compared with the unstimulated control, in agreement with parallel enzyme-linked immunosorbent assay (ELISA) quantification and qPCR analysis confirming upregulation of dopaminergic markers TH and DAT (Fig. 6E).

Wahyuni et al. [155] fabricated an rGO/multi-walled carbon nanotube (MWCNT) nanocomposite electrode via drop-casting onto a glassy carbon electrode for the simultaneous electrochemical detection of hydroquinone (HQ), DA, and uric acid (UA). Non-covalent interactions between the sp²-hybridized regions of rGO and the sidewalls of MWCNTs, reinforced by van der Waals forces, formed an interconnected 3D network that prevented rGO restacking and expanded the electrochemically active surface area [156] (Fig. 6F), with atomic force microscopy confirming a root-mean-square roughness of 70.1 nm for the composite compared to 37.0 nm for rGO alone. This structural dispersion maximized the accessibility of residual carboxyl, hydroxyl, and epoxy groups on the rGO surface, enabling each analyte to interact through a distinct mechanism, with non-covalent adsorption for HQ, electrostatic attraction for the positively charged DA, and hydrogen bonding at the amide group for UA, collectively resolving the oxidation potentials of the three analytes into well-separated anodic peaks. At the optimal 1:1 rGO/MWCNT composition (Fig. 6G), charge-transfer resistance reached a minimum of 88.2 Ω, confirming the electrochemical basis of the observed synergy. The sensor achieved linear ranges of 3-150 µM for HQ, 4-100 µM for DA, and 2–70 µM for UA (Fig. 6H-I), with limits of detection of 0.400, 0.500, and 0.300 µM, respectively, and demonstrated recovery rates of 96–103% across six human urine samples with 80.5% signal retention after seven days.

Zhang and Zhang [157] extended this structural logic further by growing ZIF-8 metal-organic framework nanocrystals in situ onto GO sheets, exploiting the residual carboxyl and hydroxyl groups on the GO surface as coordination sites for Zn²⁺ nucleation, before electrochemically reducing the composite to yield a hierarchical rGO/ZIF-8 architecture. Within this structure, rGO provides an electron-percolated conductive network that minimizes charge-transfer resistance, while ZIF-8 nanocrystals serve as rigid spacers that prevent rGO restacking and contribute a Brunauer-Emmett-Teller (BET) surface area of 1250 m²/g, collectively supplying abundant sites for thiolated DNA probe immobilization via EDC/NHS coupling. The platform was applied to label-free electrochemical detection of the KRAS G12D point mutation, a prevalent oncogenic driver in colorectal cancer, achieving a linear detection range of 100 fM to 20 nM with a limit of detection of 35 fM, a sensitivity level commensurate with circulating tumor DNA concentrations in patient blood. The biosensor discriminated the single-nucleotide mutant sequence from wild-type and mismatched controls with negligible cross-reactivity and retained 96% of its initial response after 28 days, with recovery rates of 96.8-104.5% in spiked human serum samples.

Polymer and framework hybrid composites provide important advantages for device-level biosensor engineering. Conducting polymers such as PPy can improve mechanical compatibility and support electropolymerized 3D architectures, carbon nanotubes (CNTs) can create conductive pathways and suppress graphene restacking, and metal-organic frameworks (MOFs) can introduce porosity, molecular sieving, and high surface area. However, these materials also introduce challenges. Conducting polymers may undergo swelling, dedoping, or overoxidation during long-term electrochemical operation; CNT-containing composites require careful dispersion and toxicity evaluation; and MOF-based sensors can suffer from limited conductivity, instability in aqueous media, or complicated synthesis. Therefore, rational hybrid design should consider not only analytical sensitivity but also manufacturability, biological compatibility, and long-term operational stability.

The composite strategies explored above show that the chemical interactions between GO and its functional partners govern both sensitivity and selectivity across a broad range of electrochemical biosensing applications, ranging from single-analyte tracking at living cell interfaces to multianalyte discrimination in biological fluids and ultratrace nucleic acid detection. Together with the functionalization strategies discussed earlier, these results illustrate the breadth of design choices available for tailoring GO-based electrochemical platforms to specific biosensing demands.

## Device architecture, transduction, and 3D/organoid interfaces

Although material functionalization is central to GO-based biosensor performance, material properties alone do not determine biological sensing capability. Device-level architecture strongly influences signal transduction, spatial resolution, sample compatibility, and biological perturbation. GO-based electrochemical biosensors can be configured as amperometric sensors, voltammetric sensors, impedimetric sensors, or FET-based devices, and each architecture imposes different requirements on conductivity, surface chemistry, and biological interface stability.

Amperometric and voltammetric sensors are widely used for detecting electroactive molecules such as DA, H₂O₂, UA, GSH, and glucose. These systems benefit from rGO-mediated charge transport and catalytic hybridization with metal nanoparticles or conducting polymers. Their main strengths are high sensitivity, relatively simple instrumentation, and compatibility with miniaturized electrodes. However, their selectivity depends heavily on operating potential, antifouling capacity, and the separation of overlapping redox peaks. In complex biological media, nonspecific adsorption of proteins and oxidation of interferents can alter baseline current and reduce signal stability.

Impedimetric sensors are particularly useful for monitoring binding events, cell adhesion, barrier integrity, and morphological changes without requiring direct oxidation or reduction of the target analyte. GO can improve impedimetric sensing by increasing interfacial area and providing biomolecule immobilization sites, whereas rGO can reduce charge-transfer resistance and improve signal-to-noise ratio. Nevertheless, many impedimetric biosensors rely on soluble redox probes such as [Fe(CN)_6_]^3−^/^4−^, which complicates real-time monitoring of living cells. Probe-free impedance strategies may be more compatible with long-term cell culture but often require more sophisticated interpretation of equivalent circuit parameters.

FET-based sensors transduce surface binding events into changes in channel conductance, enabling high sensitivity and rapid detection. GO can be used as a functional overlayer on graphene channels to improve antibody or aptamer immobilization density, as shown in Fig. 4A-E. However, FET sensors operating in physiological media face Debye screening limitations, especially when target binding occurs at distances greater than the Debye length from the channel surface. Therefore, probe orientation, linker length, ionic strength, and microfluidic sample handling must be carefully engineered to preserve sensitivity.

Among biological interfaces, 3D cellular models and organoids place the most demanding requirements on device design. Spheroids, hydrogel-encapsulated cells, and organoids introduce volumetric diffusion gradients, a mechanical mismatch between rigid planar electrodes and soft curved tissue, and dynamic responses that develop over minutes to days under continuous culture. Amperometric and voltammetric readouts can follow cell-derived analytes such as DA and H₂O₂ at these interfaces, whereas impedimetric readouts can track adhesion and morphological change without consuming the analyte. GO can support this transition because its oxygenated groups provide both biomolecule immobilization sites and, after partial reduction to rGO, the conductive pathways needed to preserve sensitivity within thicker biological volumes.

At living cell and organoid interfaces, electrode geometry and surface mechanics become as important as material chemistry. A planar electrode may be sufficient for monolayer culture, whereas 3D nanopillar, porous, or hydrogel-integrated architectures can improve contact with spheroids, organoids, and tissue-like constructs [158]. GO-based surfaces may promote cell adhesion through protein adsorption and nanoscale roughness, but excessive roughness, sharp edges, high oxidative content, or unstable flakes may induce cytotoxicity or inflammatory responses. Therefore, live cell GO biosensors should include viability assays, differentiation marker validation, long-term morphology tracking, and controls for potential electrochemical perturbation.

Hydrogel-integrated architecture extends electrochemical sensing from the electrode plane into the cell-laden volume, since GO embedded within a crosslinked hydrogel can be reduced in place to render the encapsulating layer electroactive. 3D nanostructured GO surfaces can also provide the high surface area and conformable contact required by curved neuronal and organoid tissues, supporting DA and oxidative-stress measurements at culture interfaces that planar electrodes cannot reach. Nevertheless, sensing within fully vascularized, perfused organoids remains difficult, because diffusion limitations, biofouling, and electrode placement constrain access to the interior of the construct [159].

Electrochemical monitoring inside organoid-on-a-chip systems is still an emerging capability rather than an established one. Most current platforms sense at the boundary of a 3D construct, whereas continuous measurement within a multi-compartment organoid under flow has yet to be widely demonstrated [160]. Standardized spheroid and organoid formats, such as conductive microwell arrays that produce uniform spheroids for electrochemical drug screening, can provide reproducible interfaces for GO-based sensors [25]. Combining engineered GO interfaces with such formats, together with multiplexed and drift-tolerant signal processing, offers a path toward long-term, quantitative, in situ measurement in organoid-on-a-chip devices [161].

## Conclusion and perspectives

GO has established itself as a versatile 2D platform for electrochemical biosensing, occupying an advantageous position between carbon-based conductivity, oxygen-rich surface chemistry, aqueous processability, and biological compatibility. The studies surveyed in this review demonstrate that GO is not a passive support but an active interfacial platform whose electronic structure, surface chemistry, and biological interactions can be deliberately engineered. Through controlled reduction, covalent and non-covalent functionalization, noble metal nanoparticle integration, and hierarchical hybridization with conductive polymers or porous frameworks, GO-based platforms have enabled sensitive detection of metabolites, neurotransmitters, oxidative stress markers, nucleic acids, proteins, and whole pathogens across diverse biological environments. Table 1 summarizes the representative platforms covered in this review, organized by functionalization strategy and detailing the target analyte, analytical performance, and detection environment for each platform.

**Table 1:** Summary of representative platforms covered in this review, organized by functionalization strategy and detailing the target analyte, analytical performance, and detection environment for each platform

Strategy	Material/Platform	Target analyte	Linear range	LOD	Detection environment	Ref.
Thermal reduction	mrUG (UGTF, 300 °C, 10s)	H₂O₂	10 µM–10 mM	6.21 µM	Buffer	[1]
Laser reduction	LRGO/anti-*E. coli* antibody	*E. coli*	917 CFU/mL–2.1 × 10⁷ CFU/mL	283 CFU/mL	Artificial urine	[79]
Electrochemical reduction	ERGO-Lys	Glucose	N/A	2.0 µM	Buffer	[87]
Electrochemical reduction	ERGO/MMP-2 aptamer	MMP-2	10 pg/mL–10 ng/mL	3.32 pg/mL	Human plasma	[91]
Covalent (Glutaraldehyde)	Laccase/GO	GSH	1 µM–100 µM	0.89 µM	Human blood	[114]
Covalent (EDC/NHS)	GO-Chi/aptamer	*HlyE* protein	1.25 ng/µL–20 ng/µL	0.137 ng/µL	Patient serum	[116]
Non-covalent (π–π)	GO/Graphene FET + antibody	SARS-CoV-2 spike/N protein	10 fg/mL–100 pg/mL	8 fg/mL	Throat swab buffer	[123]
Non-covalent (electrostatic)	GO/GDH/LPEI-Fc	Glucose	1.0–40 mM	0.28 mM	Buffer	[126]
Non-covalent (π–π + H-bond)	GO/ssDNA probe	*tet* gene	50 pM–1 nM	50 pM	River + sewage water	[127]
AuNP hybrid	SIDNEY (GO/AuNP/Au pillar)	DA	0.0122 µM–3.13 µM	29.5 nM (PBS), 7.51 nM (aCSF)	iPSC neurons + midbrain organoid	[140]
PtNP hybrid	NCI-H1975/GelMA/rGO/PtNP/rGO/SPCE	H₂O₂ (oxidative stress)	1 µM–10 µM	0.65 µM	3D lung cancer (NCI-H1975)	[143]
Au@Pt bimetallic	Au@Pt/ERGO	H₂O₂	1 mM–8 mM	0.078 mM	Buffer	[144]
PtNP heteroatom	Pt/Au-BSN-rGO	cTnI	0.1 pg/mL–50 ng/mL	0.082 pg/mL	Human serum	[145]
PdNP hybrid	PdNP/NSPGF	H₂O₂ (cell)	0.5 µM–2.0 mM	0.1 µM	Colon cancer cells	[149]
Atomically dispersed Pd	Pd-O/GO	DA	0.1 µM–50 µM	55 nM	Buffer	[150]
Polymer hybrid	GOMPON (GO/PPy nanopillar)	DA	0.5 µM–100 µM	33.4 nM	SH-SY5Y + iPSC neurons	[154]
Carbon hybrid	rGO/MWCNT	HQ / DA / UA	HQ 3-150 / DA 4-100 / UA 2–70 µM	0.400 / 0.500 / 0.300 µM	Human urine	[155]
MOF hybrid	rGO/ZIF-8	KRAS G12D mutation	100 fM–20 nM	35 fM	Human serum	[157]

A key strength of GO lies in its amphiphilic and chemically heterogeneous architecture. The coexistence of hydrophilic oxygen-containing functional groups and hydrophobic sp² graphitic domains provides multiple interaction modes, including amide coupling, imine formation, hydrogen bonding, electrostatic attraction, and π–π interaction. These properties enable GO to function simultaneously as a processable coating, a biomolecule-anchoring scaffold, a mediator of interfacial charge transfer, and a structural component in 3D or hybrid architectures. Such integrative character allows GO-based platforms to reconcile the analytical trade-offs inherent to dynamic biological monitoring, capturing both rapid transient signals and slow temporal changes within a single sensing interface. This integrative character is particularly relevant in advanced cell-based disease models, including spheroids, organoids, and organ-on-a-chip systems, where sensing interfaces must be sensitive, mechanically conformable, chemically stable, and compatible with long-term culture.

Despite these advances, the field still faces persistent challenges. Although batch-to-batch reproducibility, biofouling, and long-term operational stability still require refinement, these should be viewed as engineering opportunities. Routine reporting of oxidation state, flake size distribution, zeta potential, sheet resistance, and electrochemical impedance, together with antifouling coatings, hydrogel barriers, and microfluidic washing, will strengthen reproducibility and biological robustness. Machine-learning-assisted signal analysis may further extract reliable information from multianalyte or long-term responses. Supervised pattern-recognition models trained on labeled voltammetric datasets can classify overlapping redox signatures that conventional peak picking cannot resolve, which is especially relevant when structurally similar neurotransmitters or metabolites are released simultaneously in organoid media. In parallel, peak-deconvolution and baseline-correction algorithms can separate convolved voltammetric peaks and compensate for the progressive baseline drift introduced by electrode fouling and matrix adsorption during prolonged organoid culture, thereby supporting stable and quantitative readouts from these chemically complex environments.

Taken together, GO represents a versatile and practically adaptable material platform for next-generation electrochemical biosensing. Its amphiphilicity, chemical tunability, processability, interfacial stability, and compatibility with hierarchical hybrid architectures collectively address the core needs of biological sensing. The studies summarized in this Review demonstrate that GO-based systems have advanced beyond simple electrode modification toward sophisticated biointerfaces capable of interacting with living cells, tissues, and organoid-like structures. Looking forward, GO is expected to play a central role in the development of real-time, minimally perturbative diagnostic technologies capable of capturing dynamic biological activity with high sensitivity and functional relevance. Continued exploitation of the chemical versatility and device-level adaptability of GO will further establish GO-based biosensors as practical tools for electro-diagnostics, disease modeling, drug screening, and personalized biomedical monitoring.

## Data Availability

The datasets used and/or analyzed during the current study are available from the corresponding author on request.
